# Predicting Blood Glucose Levels with Organic Neuromorphic Micro‐Networks

**DOI:** 10.1002/advs.202308261

**Published:** 2024-04-29

**Authors:** Ibrahim Kurt, Imke Krauhausen, Simone Spolaor, Yoeri van de Burgt

**Affiliations:** ^1^ Microsystems Institute for Complex Molecular Systems Eindhoven University of Technology Eindhoven 5612 AE The Netherlands; ^2^ Max Planck Institute for Polymer Research 55128 Mainz Germany

**Keywords:** glucose prediction, hardware computing, in‐body computation, neural networks, organic neuromorphic computing, wearable

## Abstract

Accurate glucose prediction is vital for diabetes management. Artificial intelligence and artificial neural networks (ANNs) are showing promising results for reliable glucose predictions, offering timely warnings for glucose fluctuations. The translation of these software‐based ANNs into dedicated computing hardware opens a route toward automated insulin delivery systems ultimately enhancing the quality of life for diabetic patients. ANNs are transforming this field, potentially leading to implantable smart prediction devices and ultimately to a fully artificial pancreas. However, this transition presents several challenges, including the need for specialized, compact, lightweight, and low‐power hardware. Organic polymer‐based electronics are a promising solution as they have the ability to implement the behavior of neural networks, operate at low voltage, and possess key attributes like flexibility, stretchability, and biocompatibility. Here, the study focuses on implementing software‐based neural networks for glucose prediction into hardware systems. How to minimize network requirements, downscale the architecture, and integrate the neural network with electrochemical neuromorphic organic devices, meeting the strict demands of smart implants for in‐body computation of glucose prediction is investigated.

## Graphical abstract

Accurate glucose prediction is crucial for diabetic patients. Many state‐of‐the‐art prediction methods rely on artificial neural networks to offer reliable predictions, implemented as software running on conventional computing architecture. However, such software networks can be downscaled to meet the requirements of flexible, biocompatible, and low‐power neuromorphic hardware based on organic electronics. The simulated organic neuromorphic micro‐networks match state‐of‐the‐art software performance.

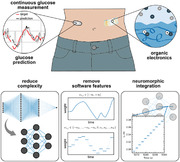

## 1 Introduction

The prediction of glucose levels is of vital importance in the management of diabetes, a chronic condition affecting millions of people worldwide.^[^ ^1^, ^2^, ^3^, ^4^ ^]^ Accurate glucose prediction allows individuals with diabetes to proactively adjust their insulin doses or dietary intake, reducing the risk of dangerous fluctuations in blood sugar levels.^[^ ^5^ ^]^ While traditional methods of glucose monitoring such as fingerstick measurements have provided valuable insights, the emergence of artificial intelligence technologies is transforming our ability to predict glucose levels with high accuracy.^[^ ^6^, ^7^ ^]^ Moreover, these advancements hold the potential to translate into implantable prediction devices toward a fully artificial pancreas, offering continuous, real‐time monitoring and management and enhancing the quality of life for individuals living with diabetes.^[^ ^8^, ^9^ ^]^

Artificial neural networks have demonstrated notable capabilities in modeling complex, nonlinear relationships within data. When applied to glucose prediction, these networks can analyze a variety of variables, including historical glucose levels, dietary patterns, physical activity, and even sleep patterns.^[^ ^7^ ^]^ By processing this diverse data set, neural networks can learn intricate patterns and correlations, ultimately enabling them to make highly accurate predictions about future glucose levels.^[^ ^10^ ^]^ The application of neural networks to glucose prediction has already shown promising results.^[^ ^11^, ^12^, ^13^, ^14^ ^]^ These models can provide patients with timely warnings of glucose fluctuations, allowing them to take proactive measures to prevent hypoglycemia (low blood sugar) or hyperglycemia (high blood sugar). Furthermore, the integration of wearable devices such as continuous glucose monitors, has facilitated real‐time data collection, improving the accuracy and responsiveness for patients and enabled data curation for neural network predictions.^[^ ^15^, ^16^ ^]^

Translating these neural network models into dedicated computing hardware would enable the realization of automated insulin delivery systems and artificial pancreases in the form of on‐body or implantable medical devices.^[^ ^15^, ^17^ ^]^ Such devices would bring significant benefits to diabetic patients, as they would no longer be reliant on wearing external devices or on manually tracking their glucose levels. Implantable prediction devices would offer a seamless and continuous monitoring solution, drastically reducing the burden on patients and enhancing their quality of life.^[^ ^18^ ^]^

The conversion of software neural networks into hardware suitable for on‐ and in‐body computation poses several challenges. Specific requirements include specialized, compact, and lightweight hardware for comfortable body integration. This hardware should exhibit low power consumption, energy efficiency, and the ability to execute neural network algorithms. Additionally, the ANN themselves should operate efficiently on this hardware, with minimal computational and memory requirements.^[^ ^19^, ^20^ ^]^ State‐of‐the‐art wearable sensor devices such as smartwatches are already widely used and allow the collection of data on a larger scale and data that would otherwise not be accessible. Evaluation of this data suggests its potential for remote and personalized healthcare and clinical applications.^[^ ^21^, ^22^ ^]^ Nevertheless, the escalation in data volume calls for more advanced processing techniques (such as machine learning) and simultaneously poses a data protection hazard. Using a localized way of data handling and processing would reduce the risks of handling sensitive health‐related data.^[^ ^23^ ^]^

Organic polymer‐based electronics present an ideal candidate to address these challenges.^[^ ^24^, ^25^ ^]^ Micro‐scale organic electronic components can efficiently mimic the synaptic connections found in biological neural networks, enabling the creation of neuromorphic systems that replicate the brain's processing capabilities similar to ANN.^[^ ^26^, ^27^ ^]^ Additionally, organic electronics operate at low voltage and are easily tunable, making them ideal for energy‐efficient hardware neural networks^[^ ^28^ ^]^ that can process information in real‐time^[^ ^29^ ^]^ within the limits of implantable devices. Simulations have already shown small‐scale, low‐voltage organic devices capable of analyzing physiological data.^[^ ^30^ ^]^

Organic polymeric materials possess several additional properties that are ideal for implantable solutions, including flexibility,^[^ ^31^ ^]^ stretchability,^[^ ^32^ ^]^ biocompatibility,^[^ ^33^ ^]^ and the ability to interface seamlessly with biological tissues.^[^ ^34^, ^35^ ^]^ When applied in implants, these materials have shown to conform to the complex and curvilinear shapes of body structures, reducing the risk of tissue damage or foreign body reactions.^[^ ^35^, ^36^ ^]^ Organic electronic materials can also be used as glucose sensors^[^ ^37^, ^38^ ^]^ or be incorporated into drug delivery systems,^[^ ^39^ ^]^ allowing for precise, controlled release of medications, an important integration step toward an artificial pancreas.

**Figure 1:**
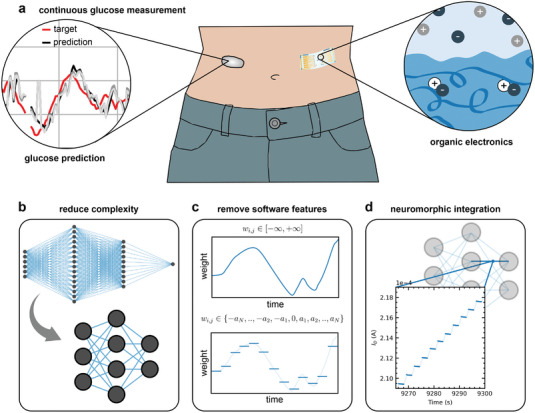
Organic neuromorphic electronics for glucose prediction. a) Continuous glucose measurement have become a vital strategy in managing diabetes. Artificial neural networks (ANNs) are used for glucose prediction. Hardware implementations of the ANNs are necessary to move towards on‐body or in‐body computation integrating seamlessly with already existing monitoring solutions. Organic electronics emulate neuronal behavior similar to that of ANNs while also being flexible, stretchable and biocompatible. They offer a great promise for small‐scale biointegration of hardware neural networks. b) Reducing the size and general complexity of the neural networks increases the feasibility of a hardware implementation. c) While pure software neural networks have a continuous weight space with no bounds, hardware devices exhibit a distinct number of conductance states within a confined range leading to limitations on the ANN weight space. d) Real‐life measurements of hardware weights are used for ANN weights.

The current state‐of‐the‐art fabrication methods for organic neuromorphic hardware do not allow the realization of (deep) hardware neural networks containing thousands of trainable weights and adopting complex architectures, typically utilized for time series prediction tasks such as glucose prediction. This study shows how hardware limitations can drive the development of efficient neural network designs, enabling the practical implementation of a glucose‐predicting neural network that can be integrated into smart wearables or implant devices (**Figure** **1**a). First, we minimize the requirements of the neural network by downscaling existing benchmark architectures without significant loss of performance. This process involves reducing the complexity of the network, and limiting memory and processing demands (Figure 1b,c). We then integrate this neural network with actual measurements from an electrochemical neuromorphic organic device (ENODe) which effectively replaces the conventional network weights directly in dedicated hardware (Figure 1d). By combining ANN optimization with the capabilities of organic electronics, we show how neuromorphic hardware can be useful to achieve in‐body computations for glucose predictions.

## 2 Results

### 2.1 Reducing Network Complexity

#### 2.1.1 Input Reduction

The landscape of glucose prediction research consists of a multitude of algorithms and predictive models, often accompanied by their own data set, making a reliable comparison difficult. With the GLYcemia Forecasting Evaluation (GLYFE, Section 4),^[^ ^12^ ^]^ a systematic review of nine machine learning models with a standardized processing pipeline (Section 4) on the same data set has been provided. Their feed‐forward neural network (FFNN, Section 4) offers a hardware‐suitable architecture^[^ ^40^ ^]^ and therefore functions as benchmark for our performance evaluation. The selected OhioT1DM dataset (Section 4)^[^ ^16^ ^]^ includes clinical data of 12 diabetes 1 patients in two Cohorts (2018 and 2020) and is publicly available to researchers. This allows a unified and freely accessible approach to evaluating glucose predictions of ANN.

Patient‐to‐patient variance is a known issue in glucose forecasting. Combined data sets of multiple individuals improves generalization by finding common features, removing person specific noise or bias and increasing the size of the data set.^[^ ^13^, ^14^ ^]^ Therefore, a combined data set is used for training and evaluation (details in Section 4).

**Figure 2:**
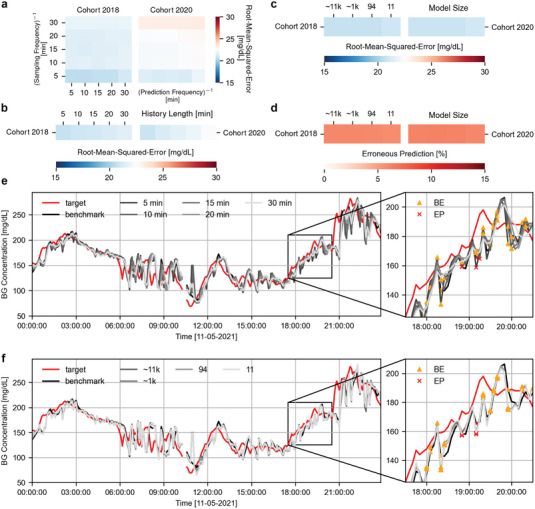
Blood glucose prediction results for reduced input and model size. a) Mean (5‐fold cross‐validation) RMSE over the individuals in Cohorts 2018 and 2020 of the OhioT1DM data set, where the models have been trained with different combinations of sampled history (180 minutes) frequencies and prediction frequencies. The results show training with alternative prediction frequencies does not effect generalization and a sampling frequency of 5 minutes has a slight advantage over the alternative options. b) Mean RMSE over the individuals in Cohorts 2018 and 2020, however, this time with a reduced history lengths that consist of two nodes only. The results show a decreasing performance when a longer duration of the history is taken over a more immediate one. c) Mean RMSE over the individuals in Cohorts 2018 and 2020, where the models have been trained with history length of 5 minutes for model architectures [128, 64, 32, 16] (11265 parameters), [48, 16] (945 parameters), [9, 6] (94 parameters) and [2, 1] (11 parameters). The results show no loss of RMSE with reduction of model size except for the smallest model. d) The percentage of erroneous predictions for the models described in (c), which show likewise to the RMSE that the number of erroneous predictions does not increase with the reduction of model size.(e) Example of the real‐time differences between the blood glucose predictions for the models described in (b), with target (red) the target data for individual 563 on the day 8 of the test set, benchmark (black) the predictions for the base model with 180 minutes of history sampled at 5 minutes. The results show larger time lags and increasingly smooth transitions with less immediate gradients, which positively effect the CG‐EGA evaluation metrics but not the RMSE. f) Example of the real‐time differences between the blood glucose predictions for the models described in (c) and (d), for the same test day and individual as in (e). The results show little to no differences among both the base predictions as well as all the predictions made by the smaller models.

With regard to glucose prediction, blood glucose, insulin, and meal data are most frequently selected input features in the literature.^[^ ^11^ ^]^ This is primarily due to possible preemptive cues meals and insulin injections are able to provide, reducing the time lag of potential blood glucose peaks or valleys. With consistent and accurately reported data, the triplet of input features outperform an identical model trained on blood glucose only. However, despite adding to the performance of many models in the literature, the predictive gain remains marginal. It is in the interest of minimizing the footprint of the required network and the removal of any sensitive and invasive input features that the single feature considered in this work is blood glucose. The OhioT1DM blood glucose data is sampled every 5 min and by default any history length selected as the input is sampled with the same frequency. To assess the significance of the sample count in the history with the intent to minimize the number of input nodes into the network, different sampling frequencies (one sample every *n* minutes) are investigated (**Figure** **2**a). Furthermore, by altering the sampling frequency a minimal form of filtering is applied due to the inherent smoothing of the reduced number of sampling points. The FFNN architecture along with all training hyperparameters are described in Section 4 and the evaluation metrics are explained in Section 4. The blood glucose history length of 180 min is sampled with 5, 10, 15, 20 and 30 minutes respectively. Furthermore, as an additional assessment of the effect of down‐sampling of the dataset, a range of prediction frequencies are also investigated. By changing the prediction frequency (one prediction every *n* min), the training is performed on increasingly sparse data, adding to the models generalization. As an example, a prediction frequency of 30 min (one prediction every 30 min) subjects the model to a six times smaller dataset during training without the complete exclusion of the data of five individuals. It increases the intervals in the time‐series data to improve efficiency and remove unnecessary details. To note, a sampling and prediction frequency of 5 min provide an identical iteration to the GLYFE (Section 4), but in our study with blood glucose as single input feature and a combined data set. We refer to this as benchmark. An overview of the proposed input parameters and frequency definitions are provided in Figure S1 (Supporting Information).

The Root‐Mean‐Squared‐Error (RMSE) evaluation of this research predominately falls within the range of 15–30 mg dL^–1^, this range is kept constant throughout all figures for comparability. The investigated sampling and prediction frequencies do not show to improve on the RMSE of the cross‐validation models of the considered architecture (Figure 2a). On the contrary, a similar performance is maintained over all prediction frequencies and a sampling frequency of 5 min shows to have a slight advantage over all alternative options. Additional evaluation metrics, such as the continuous glucose error grid analyses (CG‐EGA) metrics (Section 4) and time lag are provided in Figure S2 (Supporting Information). The results depicted in Figure 2a are not definitive enough to establish that a sampling frequency of 5 min is favorable. A distinction should be made whether the model performance is rooted in an increased number of sampling points in the history caused by a higher sampling frequency or if this stems from providing a more recent gradient by having less distance between neighboring points. To investigate this, we train networks with a history length consisting of two samples only. The second sampling point lies 5, 10, 15, 20, and 30 min in the past to assess the effect of a longer averaged‐out gradient (30 min) to a more recent, immediate one (5 min). The results in Figure 2b and Tables S1 and S2 (Supporting Information) depict a decreasing performance with an increasing two‐point history length. This confirms that the model's performance relies mainly on short‐term gradients.

Most interestingly, we see little change in the overall performance even though the input information has been drastically reduced from three to one features and from 36 to 2 sampling points in time. The mean RMSE over the test sets with only a history length of 5 min is 20.74 mg dL^–1^ over Cohort 2018 and 20.83 mg dL^–1^ over Cohort 2020. The benchmark performance with 180 min of history has a mean RMSE of 20.72 and 21.43 mg dL^–1^ over the respective Cohorts. In contrast, the RMSE of the GLYFE over Cohort 2018 constituted to 20.65 mg dL^–1^ with blood glucose, meal data and insulin as the input features (reproduced with the combined dataset). This demonstrates not only replication of performance but also a slight improvement compared to the same model with personal datasets as presented by the GLYFE originally (RMSE of 21.00 mg dL^–1^). We hypothesize that is due to a more significant influence and information content of the most recent gradient and blood glucose value.

Figure 2e shows an exemplary one‐day excerpt of the predictive performance and the underlying differences between the two‐point history lengths in individual 563. The discussed smoothing of the prediction due to extending the history length is visible here. A shorter history representing a more recent gradient has a lower RMSE and leads to more adaptive predictions with harsher swings. The inset of Figure 2e provides a closer look at how predictions are categorised based on the CG‐EGA evaluation metric with benign errors as yellow triangle and erroneous predictions as red cross. Accurate predictions are unmarked. The benchmark model (adapted FFNN of GLYFE), depicted in black, causes more erroneous predictions in comparison to the iterations with a long history (10–30 min length), but shows a lower RMSE and time lag (Figure S3e, Supporting Information). This is accredited to the harsher swings that occur when a shorter history time frame is considered. Even though these predictions follow the target line more accurately, the rate (gradient) differences are sufficiently jagged to cause the CG‐EGA evaluation metric to categorize the predictions as erroneous. A full overview of CG‐EGA metrics is provided in Figure S3 (Supporting Information). Despite better performance in the CG‐EGA metrics, the favor is given to training iterations with a lower RMSE. Improved performance in the clinical evaluation metric CG‐EGA is not necessarily rooted in a conclusive performance advantage but can also be caused by a metric‐related limitation. Section 2.1.3 provides more details on this limitation.

Decreasing the history length to a two‐point gradient, and selecting blood glucose as the single input feature reduces the number of parameters in the network from the original 24 883 to 11 265 (≈11 k) without significant loss of performance (20.74 mg dL^–1^ compared to 20.65 mg dL^–1^ over Cohort 2018). This reduced input not only entails lower energy demands for computation but also minimizes invasive data collection for future patients.

#### 2.1.2 Size Reduction

Since significant input cuts did not cause performance drops this indicates that the network and information complexity are not matched yet and further size reduction might be possible without compromising the model accuracy.

Figure 2c and Figure S4 (Supporting Information) show the performance of the model for a range of different model sizes, more specifically, for hidden layer sizes [128, 64, 32, 12], [48, 16], [9, 6], and [2, 1] that correspond to 11265 (≈11 k), 945 (≈1 k), 94, and 11 parameters, respectively. As hypothesized, the required complexity for the problem of glucose prediction with benchmark performance is minimal. No significant performance loss is observed with a size reduction of multiple orders of magnitude (Tables S1 and S2, Supporting Information). Only a network size of 11 shows a slight decrease in performance (RMSE of 20.96 mg dL^–1^ over Cohort 2018). This is remarkable, as a model with only 94 parameters is able to perform equally well as the model that is over two orders of magnitude larger. A closer look at sample to sample differences, depicted in Figure 2f, reveals minimal observable differences.

Figure 2d shows the percentage of the erroneous predictions according to CG‐EGA for the same subset of model sizes. Likewise to the RMSE, the number of erroneous predictions does not increase with the reduction of the network size. The percentage of erroneous predictions over the Cohort of 2018 is 6.03% (now with a reduced input history length and number of features), this is slightly higher than the percentage of erroneous predictions for the benchmark model, which equalled 4.80% over the Cohort of 2018. This increase despite being unfavorable is a trade‐off worth considering, as the reduction in model size and input is substantial compared to the accuracy decrease. Moreover, as explained earlier, this performance decrease in the CG‐EGA evaluation metric is primarily rooted in the blood glucose rate differences. Due to this effect, a small improvement is observed in the the smallest sized model (5.77% over Cohort 2018). As previously stated, this behavior stems from smoothing of peaks and jagged gradients for the predictions in Figure 2e,f and also seen from the zoom‐ins.

#### 2.1.3 Removing Software‐Specific Features

**Figure 3:**
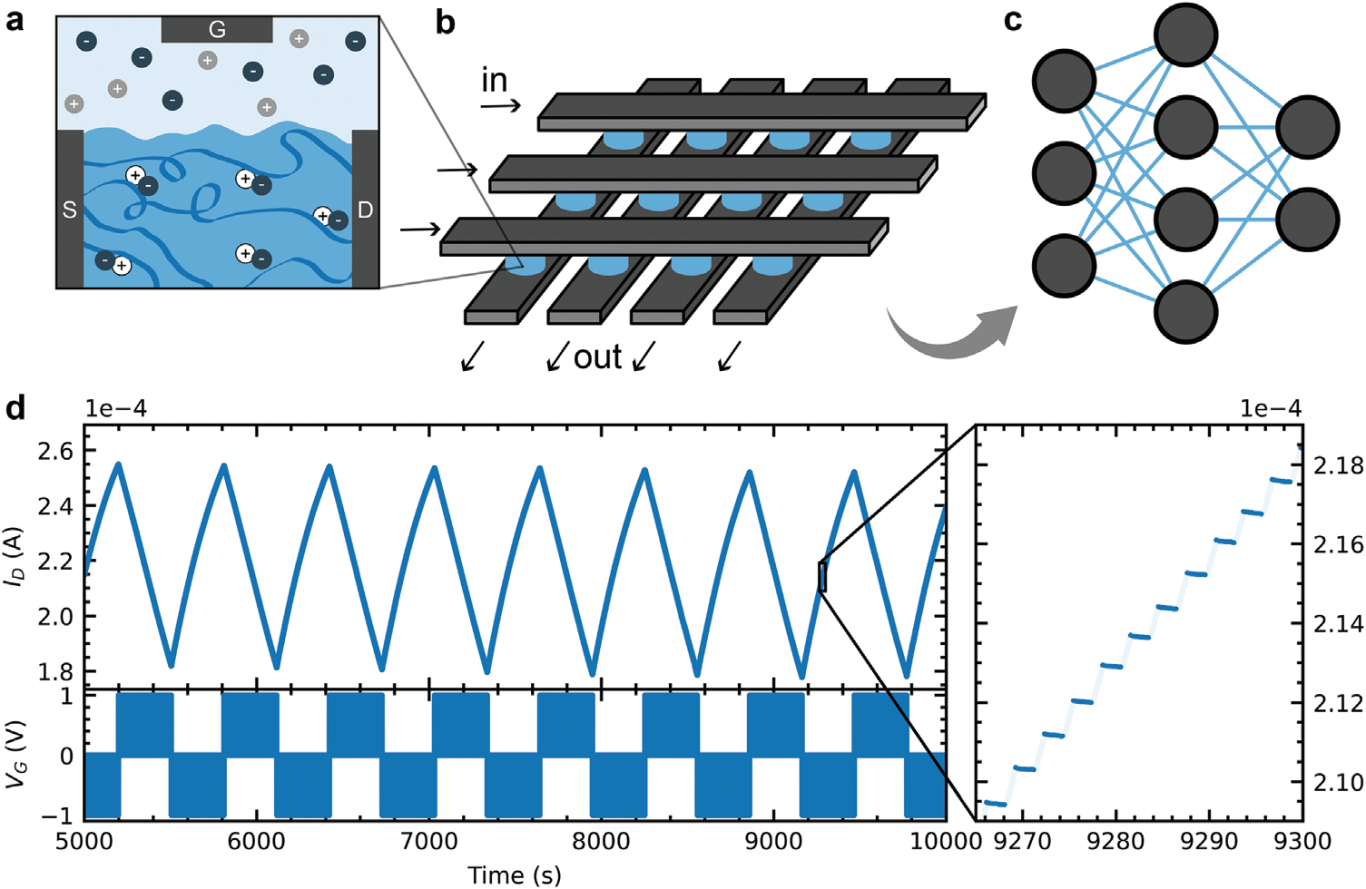
ENODe measurement. a) The electrochemical neuromorphic organic devices consists of three terminals: source (S), drain (D) and gate(G). Source and drain electrode are connected with the p‐type polymer PEDOT:PSS. An electrolyte provides an ionic connections to the gate. b) The ENODes can be integrated in larger‐scale architectures like crossbar arrays. c) Each device then represents on weight inside a hardware neural network. d) A measurement of 100 conductance states shows high linearity in behavior and stability of time.

To understand how we can further simplify the prediction networks, we first need to understand the physical devices for our hardware setup. The ENODe has three terminals: source, gate and drain (**Figure** **3**a, dark grey). Source and drain electrode are connected via the organic polymer PEDOT:PSS (poly(3,4‐ethylenedioxythiophene) polystyrene sulfonate) operating as channel (Figure 3a, dark blue). Details on device fabrication are provided in Section 4 and device architecture is pictured in Figure S7 (Supporting Information). The gate voltage controls the conductance of the channel material through movement of ions between the channel material and the ion‐containing electrolyte (Figure 3a, light blue). By integrating multiple ENODes into a crossbar configuration (Figure 3b) it is possible to translate network architecture into hardware.^[^ ^28^ ^]^ Each weight of the software neural network is represented by an ENODe in the hardware setup (Figure 3b,c in blue).

A substantial reduction of the network complexity and the amount of states that its weights can assume, increases the feasibility of a present day hardware implementation. However, all models that have been trained and evaluated so far employ training methods difficult to implement in hardware (e.g., the Adam optimizer), and unbounded, floating‐point numbers to implement weights' values. To determine whether a hardware application is feasible, a more realistic training environment as detailed in **Table** **1** is considered. The Adam optimizer is removed and no additional moments in the optimizer are added. Furthermore, the number of states for the neural network weights are bounded and discretized as the active materials in ENODes have a restricted on/off ratio and a fixed number of distinct stable states. Figure 3d showcases a characteristic measurement of the ENODe's neuromorphic behavior. Measurement details are provided in Section 4 and the measurement setup is shown in Figure S7a (Supporting Information). We can access 100 distinct conductance states with highly linear set and reset behavior and stable state retention (zoom‐in of Figure 3d). These 100 conductance states are extracted from the measurement and mapped into a bounded parameter space for the weights, conserving the set and reset linearity during the mapping. Depending on the direction of the weight update, we round to the closest possible state in the set or reset selections. Moreover, the SELU activation function is replaced with a ReLU activation function as exponentiation in hardware is currently unfeasible.^[^ ^41^ ^]^ This alteration does not effect the performance as the SELU activation function loses its advantage over a ReLU for shallow networks.^[^ ^42^ ^]^ Lastly, batch training is removed as parallel processing of samples is not attainable following circuit law.

**Table 1:** Comparison between the hyperparameters of a software environment and a realistic hardware environment. Using ENODe measurements, 100 stable states are selected as weight and bias values in respective set and reset selections. Other training configurations, such as the optimizer, activation functions and batch learning method, are replaced with hardware feasible alternatives.

Hyperparameter	Software	Hardware
Activation function	SELU	ReLU
Weight states	10^19^ (floating‐point)	100 (conductance)
Optimizer	Adam	None
Batch size	1500	1

### 2.2 Neuromorphic Integration

The hardware simulations are performed using three different parameter spaces. Next to the 100 ENODe states introduced earlier, linearly‐spaced arrays of 1000 and 100 states are also considered. These linear spaces validate whether any performance loss is caused by non‐linearities in the device data or by the bounded number of states. The introduction of an upper and lower limit of the network weights, namely the parameter bounds, adds an additional hyperparameter. These bounds have to be carefully selected as they drastically affect the models ability to converge to an optimum. Narrowly selected bounds directly limit the parameter space, causing premature convergence to under‐performing solutions during training. Widely selected bounds increase the step size between states, reducing training precision and increase the likelihood of convergence to local minima. Here, the maximum and minimum weight value of the previous (unbound) model are chosen as upper and lower bounds respectively (**Table** **2**). A more studious investigation of this hyperparameter could further improve the model performance.

**Table 2:** Parameter bounds for linearly‐spaced network weights and ENODe‐extracted weights.

Model size	Boundaries
[48, 16]	−1.2, 1.2
[9, 6]	−1.5, 1.5
[6, 3]	−1.7, 1.7
[4, 3]	−1.7, 1.7
[2, 1]	−2, 2

**Figure 4:**
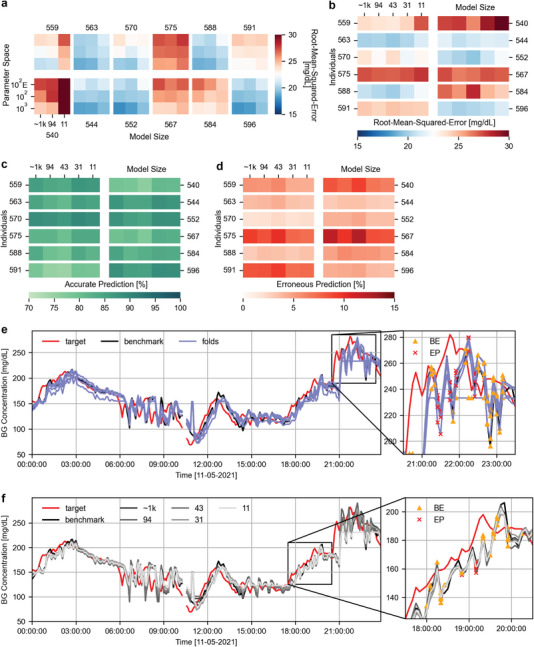
Blood glucose prediction results for hardware simulated models (ENODe). a)Mean RMSE over the test sets of all individuals in the OhioT1DM dataset, where the model has been trained with hardware feasible hyperparameters and a line‐space of a 1000, 100 and 100 ENODe states (from measurements). The results show decreasing performance with the introduction of fewer and less linear states. b) Mean RMSE over the test sets of all individuals in the OhioT1DM data set, where the models have been trained with hardware feasible hyperparameters (Table 1, Hardware) and ENODe characteristic parameter selections. The results show inconsistent performance of the models with decreasing size. This is accredited to how well the hyperparameters are tuned, as for model [9, 6] the hyperparameters are better optimized. c) The percentage of accurate and erroneous (d) predictions for the models (and similar folds) provided in (a). Results show slightly better performance for the sub optimally tuned models, this is due to the inherit property of the CG‐EGA to penalize blood glucose rate differences more severely than value differences. e) Example of the real‐time differences between the blood glucose predictions of the 5 models of the cross‐validation folds of model [4, 3], with target (red) the target data for individual 563 on the day 8 of the test set, benchmark (black) the predictions for the benchmark model with 180 minutes of history sampled at 5 minutes (software) and folds (purple) the cross‐validation folds. The results show that folds are prone to be capped in their glucose level reach. Nonetheless, does at least one fold convergence adequately. f) Identical example to (e) however this time the converged folds of the each of the hardware simulated sizes are presented with the exception of model [2, 1] (has no fully converged folds). Depending on how well the folds are optimized, is significant performance loss with hardware networks preventable.

**Figure** **4**a and Figure S5 (Supporting Information) show the performance for different parameter spaces at varying model sizes. With the introduction of less sophisticated training elements (proper) model convergence becomes more challenging due to the strong limitations imposed by hardware. We note that this could be circumvented by further hyperparameter optimization and/or selecting a suitable, non‐random initialization seed. Hyperparameter tuning becomes increasingly difficult for smaller networks, as the window for proper convergence and precision might not overlap with the required parameter bounds to cover the full spectrum of blood glucose levels. It is clear from the results in Figure 4a that performance decreases (Tables S3 and S5, Supporting Information) with the introduction of limited parameter states and non‐linearity in the selection spectrum, but again, this is highly dependent on how well the hyperparameters are optimized. Excluding the smallest model (11 parameters), model sizes 94 and ≈1k have at least one fold that performs adequately and is able reach the complete range of blood glucose level. Figure 4a also depicts that for the smallest network size the RMSE performance drops drastically (24.28 mg dL^–1^ over Cohort 2018). Further investigation into the smallest network confirms that none of the 5 folds converged optimally and in turn were not able to reach every glucose level for any of the parameter spaces (Figure S8, Supporting Information, for ENODe parameter space) showing that there are clear limitations for downsizing but at extreme level only.

Model [9, 6] (94 parameters) shows acceptable performance with an ENODe parameter space (22.97 mg dL^–1^ over Cohort 2018) and therefore the evaluation is extended with two additional network sizes [6, 3] (43 parameters) and [4, 3] (31 parameters), depicted in Figure 4b and Figure S6 (Supporting Information). We choose a fixed initialization for models of size 43, 31, and 11 to ensure convergence of all 5 folds despite heavy impositions, but provide the results of the standard fivefold cross validation in Figure S9 (Supporting Information). The difference of performance is another clear indicator that hyperparameter selection and initialization are pivotal for performance at this size. The model of 94 parameters outperforms other model sizes. This is however again considered to be caused by superior hyperparameter selection, because the plain software models depicted in Figure 2 have already demonstrated how well the performance can be maintained even for the smallest network considered.

The performance of the 31‐parameter model run on the ENODe parameter space is depicted in Figure 4e for all five folds with seemingly little deviation from the benchmark. As explained previously, proper model convergence is challenging. One fold is not able to reach the full height of blood glucose values and cuts off (Figure 4e, zoom‐in). Noticeably, when considering the CG‐EGA evaluation metrics, the remaining converging folds as well as the benchmark seem to cause more frequent erroneous predictions (red crosses in zoom‐in) than the fold that is capped and therefore show zero rate change. The converging fold follow the true blood glucose level more closely, however, the jagged trend of the rate change is penalized more severely by the CG‐EGA evaluation leading to worse performance according to this metric. Therefore, the CG‐EGA metric should never be considered as the sole figure of merit.

Figure 4f depicts the same exemplary excerpt as in Figure 4e, showcasing one converging fold of all model sizes (with fixed initialization for models of size 43, 31, and 11). As mentioned before, the smallest model (11 parameters), similar to the software environment model, is cut off in all training folds (Figure S8, Supporting Information). The model with 94 parameters appears to perform better than any of the smaller or larger sized models with the same limitations. However, this can not be clearly accredited to its size but rather our ability to tune the model optimally. It is expected that the other models (excluding the 11‐parameter model) are also able to reach the similar performance to their plain software twin with a more vigilant tuning of the hyperparameters such as the parameters bounds and learning rate.

The performance of the ENODe simulated networks using the CG‐EGA evaluation metrics is shown in Figure 4c,d. The 94‐parameter model appears to perform worse in both the percentage of accurate and erroneous predictions, however, the more closely the model follows the benchmark performance (more jagged) the more severe it is penalized in the CG‐EGA evaluation. For the 31‐parameter network, the number of erroneous predictions is 4.55% over Cohort 2018. This is close to the the benchmark model with roughly ≈ 11 k parameters (4.80%). The percentage of accurate predictions for the same model is 84.85% over the same Cohort (compared to 80.4% for the benchmark model).

This means that we can perform an extreme miniaturization of the benchmark model and include limitations due hardware‐based systems without significant loss of performance. This truly remarkable miniaturization of neural networks for blood glucose prediction meets the demands for implantable devices or on‐body computation.

## 3 Discussion and Outlook

This study demonstrates the feasibility of redesigning ANNs while carefully accounting for hardware constraints. This approach allows for hardware systems capable of achieving state‐of‐the‐art performance in blood glucose prediction. Moreover, these systems are suitable for implementation in both on‐body and implantable devices, offering promising prospects for redefining diabetes management.

In particular, our work successfully demonstrates the feasibility of minimizing neural networks to reduce network complexity, memory consumption, and processing demands, ultimately narrowing down input data to a single feature–blood glucose measurements. Remarkably, this approach shows no significant loss in performance. Furthermore, our results show that networks of organic neuromorphic devices maintain benchmark‐level performance, despite the challenges posed by bounded parameter spaces and non‐linearities.

This marks a substantial step towards the development of implantable, on‐body, or wearable systems for glucose prediction and diabetes management. As this research focuses on reducing the footprint of neural networks while maintaining performance, future work needs to look into further optimization and the development of practical implementations. Addressing the challenges of hardware adaptation, including fine‐tuning parameter bounds, long‐term stability and examining the scalability of these models across a wider patient population will be critical. Additionally, exploring the integration of organic polymer‐based electronics and neuromorphic systems in practical devices, with a focus on energy efficiency, biocompatibility, will be a promising direction. Organic electronics could not only be used for computing, but also for sensing and drug delivery allowing monolithic integration of the complete system. Ultimately, the translation of these findings into real‐world solutions holds the potential to transform diabetes management by providing individuals with more accurate, continuous, and minimally invasive glucose monitoring, reducing the burden on patients, and improving their overall quality of life.

## 4 Experimental Section

### GLYFE

The GLYFE (GLYcemia Forecasting Evaluation) provides a benchmark of the nine different data‐driven models in the field of glucose prediction,^[^ ^12^ ^]^ evaluated on the OhioT1DM data set (Section 4). Using this publicly available data set allows for more consistent comparisons between established architectures. The GLYFE provides the performance of a Feed‐Forward Neural Network (FFNN) evaluated on the individuals of Cohort 2018. This FFNN has a model size [128, 64, 32, 16] and uses blood glucose, carbohydrate (meal) and insulin data as its input leading to a network of 24833 parameters. The architecture uses an *Adam* optimizer, SELU activation functions, mini‐batch learning (1500 per batch), a Mean‐Squared Error loss function, a logarithmic hyperparameter search over the learning rate within [10^−4^, 10^−2^] and early stopping with a patience of 100 epochs. The evaluated iterations can be categorized as single or individual models, the data of a single individual is used for his/hers personal model only (data not shared across single models). The RMSE over the individuals of Cohort 2018 using the personal FFNN models was denoted to be 21.00 mg dL^–1^.^[^ ^12^ ^]^ As combined data sets improve neural network generalization, the training sets of the patients in Cohort 2018 were combined into a single data set to improve the model abstraction, each patient attributing to ≈17% of the data. The training sets of the Cohort 2020 patients were excluded from the training, only the test sets were considered. This was with reference to future real‐time personalization efforts, which hardware neural networks could provide. Moreover, it provided an overview of how well models perform on patients that have provided data to the training effort and patients who have not. All iterations and models were trained with this combined data set.

### Preprocessing

The continuous glucose monitoring data in the OhioT1DM data set contains many interruptions accredited to device malfunctions or user errors. To set up a constructive comparison to the GLYFE,^[^ ^12^ ^]^ an identical pre‐processing pipeline was set up. Linear interpolation of the glucose readings was performed on samples with two known neighbors. Linear extrapolation of the glucose readings was performed when linear interpolation was not possible. All samples for which the ground truths were not known were disregarded. Furthermore, fivefold cross‐validation was applied on the training set following a 80/20 percent distribution. Early stopping was applied during training to improve generalization. Finally, feature scaling in the form of standardization was performed to ensure consistent data distributions across the partitioned data sets.

### Network Architecture

The FFNN networks trained in paragraph 2.1.1 with architecture [128, 64, 32, 16] use an Adam optimizer, SELU activation functions, a batch size of 1500 and is trained for a maximum of 2500 epochs with early stopping with a patience of 100 epochs and a logarithmic learning rate search within [10^−4^, 10^−2^], identical to the configuration used by [12] elsewhere. Depending on the input features selected and the length of the history, the input size ranges from 108 to 2 nodes. The output is all times a single regression node that represents the future glucose level. For the models described in Section 2.1.2 this was equivalent, other than the reduction of the history length from 180 min to the respective lengths. For the models described in Section 2.1.3, the Adam optimizer was removed, the SELU activation function was replaced by a ReLU activation function, the batch size was set to 1, and the learning rate search window was extended to be within [10^−5^, 10^−1^]. The model was trained for 10 epochs with no early stopping and the best performing iteration was selected as the evaluated model.

For the adjusted parameter space. the ENODe measurements or line‐spaces were mapped between the selected parameter bounds while maintaining device linearity. Two different mapped selection were set up, based on the set or reset operation of the device. Depending on the sign of the parameter update gradient, the parameter was rounded to the closest value in the respective selection.

The parameter bounds for different model sizes were specified in Table 2. All code was written using Python (3.7.16) and Pytorch (1.13.1) for the machine learning framework and is available upon request.

### OhioT1DM Data Set

The OhioT1DM data set^[^ ^16^ ^]^ is a publicly available clinical data set that aims to promote and foster the development of glucose prediction algorithms. The data set contains the information of 12 patients with type 1 diabetes on insulin pump treatment and includes blood glucose data sampled every 5 min by continuous glucose monitoring, fingerstick glucose measurements, basal insulin rates, temporary basal insulin rates, bolus insulin injections, meal‐, sleep‐, work data, stress, hypoglycemic events, illness, exercise, heart rate, galvanic skin response, skin temperature, air temperature, number of steps (Cohort 2018), and acceleration data (Cohort 2020) of each individual. The total training and test sets consisted of 8 weeks, where the last 10 days were considered as test set. A distinction was made between the Cohorts of 2018 and 2020, as the data set was updated with the latter six individuals in 2020. Apart from the different diagnostic tools used for measuring physical activity data, other data features matched for all individuals. The patients privacy was protected by use of personal identification numbers (PID) and all data were fully de‐identified according to the Safe Harbor method. The six patients of Cohort 2018 had PID 559, 563, 570, 575, 588, 591 and patients of Cohort 2020 had PID 540, 552, 567, 584, and 596.

### Evaluation Metrics

The FFNN model of the GLYFE^[^ ^12^ ^]^ that is used as benchmark performance in this study is evaluated using the Root‐Mean‐Squared‐Error (RMSE), as well as the continuous glucose error grid analyses (CG‐EGA). The CG‐EGA provides insight into the clinical accuracy of blood glucose predictions, and provides a more critical assessment of predictions made in each of the glycemia ranges (hypoglycemia, euglycemia, and hyperglycemia).^[^ ^43^ ^]^ It categorizes a prediction to be either accurate, benign or erroneous based on the combined contribution of value and rate difference between the prediction and the true target. Accurate predictions are the optimal classification, benign errors are accredited to predictions that are inaccurate but do not bear any severe clinical consequences. Erroneous predictions, on the other hand, are inaccurate prediction that could cause life threatening complications.

### Device Fabrication

Standard microscope glass slides (75 mm × 25 mm) were cleaned in a sonicated bath, first in soap solution (Micro‐90 (Sigma‐Aldrich)) and then in a 1:1 (v/v) solvent mixture of acetone and isopropanol. Gold electrodes for source, drain, and gates were photolithographically patterned with negative photoresist AZ nLof2035 (MicroChemicals) and AZ 726MIF Developer (MicroChemicals) on the cleaned glass slides. A chromium layer was used to achieve better adhesion of the gold. The photolithography foil masks were designed using KLayout^[^ ^44^ ^]^ and the complementary pypi‐package koala.^[^ ^45^ ^]^ Each glass slide contained 12 devices with fixed dimensions. The channel dimensions of the neuromorphic device were as follows: L = 400 µm and W/L = 2 with a lateral gate of the size 1000 µm by 1000 µm and 150 µm distance between the gate and the channel. The complete layout is depicted in Figure S7 (Supporting Information). Two layers of parylene C (Specialty Coating Systems (SCS) coatings) were deposited. Soap (Micro‐90 soap solution, 2% (v/v) in deionized water) was used for separation between the layers, allowing the peel‐off of the upper layer. An adhesion promoter (silane A‐174 (γ‐methacryloxypropyltrimethoxysilane) (Sigma–Aldrich)) was added to the lower layer of parylene C to prevent detachment. This layer insulated the gold electrodes. In a second photolithography step with positive photoresist AZ 10XT (MicroChemicals) and AZ Developer (MicroChemicals), the channel and lateral gate dimensions of the devices were defined. Reactive ion etching with O2 plasma was used to carve out the channel and corresponding gates.

Before preparing the polymer solution, PEDOT:PSS (Clevios PH1000, Ossila) was sonicated for 30 min. The polymer solution contained: 94 vol% PEDOT:PSS, 4.9 vol% ethylene glycol (Merck), 1 vol% GOPS (Merck), and 0.1 vol% DBSA (Merck). The soultion was filtered through a 0.45 µm PES filter and spincoat. The device was baked at 120 °C for 1 min. The sacrificial upper parylene C was peeled off to confine the polymer inside the gate and channel regions. It was hardbaked at 140 °C for 1 h to ensure proper cross‐linking. Excess soap was rinsed off with de‐ionized water and the device was stored in DI water overnight. One hundred microliter phosphate‐buffered saline was dropcasted as electrolyte.

### Electrical Characterization

For measurements of the electrical characteristics of volatile and non‐volatile devices, a Keithley 2602B SourceMeter was used. The source measure units at the three device terminals were connected with needle probes the measurement system (see Figure S7, Supporting Information). For non‐volatile measurements of the ENODe, a mechanical switch in series with a resistance *R*_G_ = 100 MΩ was added between the gate of the device and the measurement system and enhanced the analog memory phenomena. The switch forced open‐circuit potential condition between the gate and channel, while the gate resistor *R*_G_ downscaled and limited the gate current in the range of nanoamperes.

## Conflict of Interest

The authors declare no conflict of interest.

## Supporting information

Supporting Information

## Data Availability

The data that support the findings of this study are available from the corresponding author upon reasonable request.
