# Supplementary material for: Predicting Blood Glucose Levels with Organic Neuromorphic Micro‐Networks
Source: Adv Sci (Weinh). 2024 Apr 29;11(27):2308261. doi: 10.1002/advs.202308261 (PMC11251550; doi:10.1002/advs.202308261)
Supplement: Supplementary file 1 — Supporting Information [file ADVS-11-2308261-s001.pdf]

ADVANCED SCIENCE
Open Access

# Supporting Information

for *Adv. Sci.*, DOI 10.1002/advs.202308261

Predicting Blood Glucose Levels with Organic Neuromorphic Micro-Networks

*Ibrahim Kurt, Imke Krauhausen, Simone Spolaor and Yoeri van de Burgt**

**Supplementary Materials for**

# Predicting blood glucose levels with organic neuromorphic micro-networks for in-body computation

*Ibrahim Kurt*[1†]

*Imke Krauhausen*[1,2†]

*Simone Spolaor*[1]

*Yoeri van de Burgt*[1*]

[1] Microsystems, Institute for Complex Molecular Systems, Eindhoven University of Technology, The Netherlands

[2] Max Planck Institute for Polymer Research, Mainz, Germany

[†] equal contribution

*corresponding author: y.b.v.d.burgt@tue.nl

***This PDF file includes:***

- *Supplementary Figures S1-S9*
- *Supplementary Tables T1-T4*

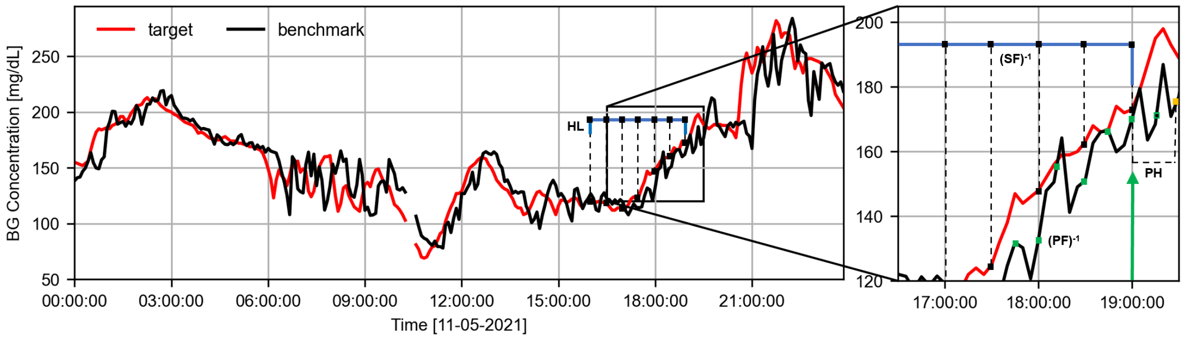


**Figure S1. Overview of the input parameters and frequency definitions.** The history length (HL) defines the range (blue) of blood glucose history used as the input for the model, here 3 hours of blood glucose history is indicated with respect to current position in time (green arrow). The sampling frequency (SF) defines the number of sampling points selected from the defined history length (black), expressed in one sample every n minutes. Here, for illustrative purposes, sampling period ($SF^{-1}$) is selected to be 30 minutes. Similarly, the prediction frequency (PF) defines the number of predictions made every n minutes (green), the greater the value for n, the sparser the training data becomes, prediction period ($PF^{-1}$) is illustrated for n = 15 minutes. Predictions are made for a specific prediction horizon (PH), namely 30 minutes (yellow), this horizon is fixed in this dispensation.

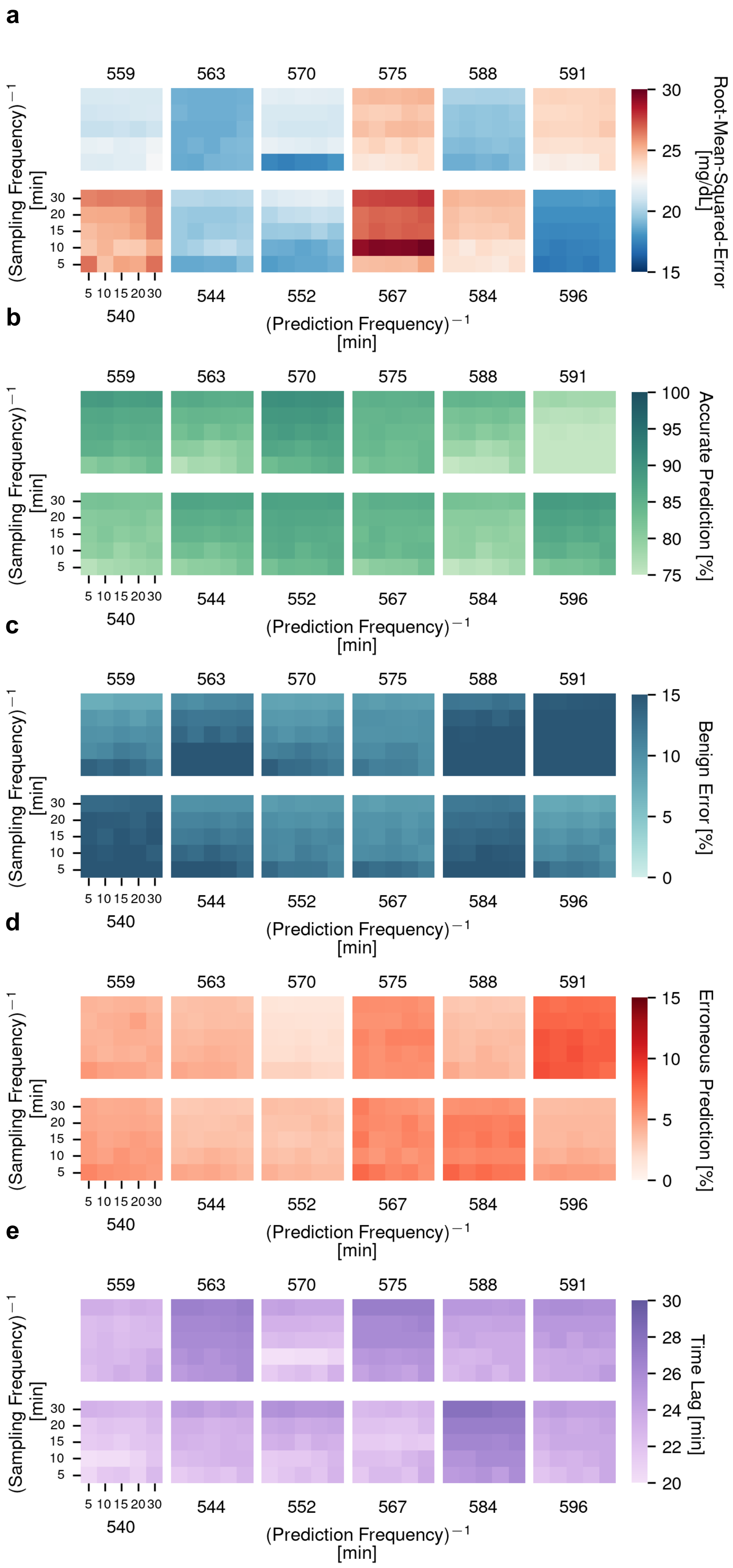
a
559
563
570
575
588
591
(Sampling Frequency)$^{-1}$ [min]
30
20
15
10
5
5 10 15 20 30
540
544
552
567
584
596
(Prediction Frequency)$^{-1}$ [min]
30
25
20
15
Root-Mean-Squared-Error [mg/dL]
b
559
563
570
575
588
591
(Sampling Frequency)$^{-1}$ [min]
30
20
15
10
5
5 10 15 20 30
540
544
552
567
584
596
(Prediction Frequency)$^{-1}$ [min]
100
95
90
85
80
75
Accurate Prediction [%]
c
559
563
570
575
588
591
(Sampling Frequency)$^{-1}$ [min]
30
20
15
10
5
5 10 15 20 30
540
544
552
567
584
596
(Prediction Frequency)$^{-1}$ [min]
15
10
5
0
Benign Error [%]
d
559
563
570
575
588
591
(Sampling Frequency)$^{-1}$ [min]
30
20
15
10
5
5 10 15 20 30
540
544
552
567
584
596
(Prediction Frequency)$^{-1}$ [min]
15
10
5
0
Erroneous Prediction [%]
e
559
563
570
575
588
591
(Sampling Frequency)$^{-1}$ [min]
30
20
15
10
5
5 10 15 20 30
540
544
552
567
584
596
(Prediction Frequency)$^{-1}$ [min]
30
28
26
24
22
20
Time Lag [min]

**Figure S2 (previous page). Additional evaluation metrics on the analyses of different combinations of sampling frequency (180 minutes history) and prediction frequency**. Mean (5-fold cross-validation) (a) RMSE, percentage of (b) accurate predictions, (c) benign errors, (d) erroneous predictions and (e) time lag for each combination of sampling frequency and prediction frequency evaluated on the test sets of all individuals in the OhioTD1M dataset. The CG-EGA evaluation metrics slightly disfavor smaller sampling frequencies, however, the time lag similarly to the RMSE slightly favors small sampling frequencies. A larger prediction frequency does not show to have any advantage over the default of 5 minutes.

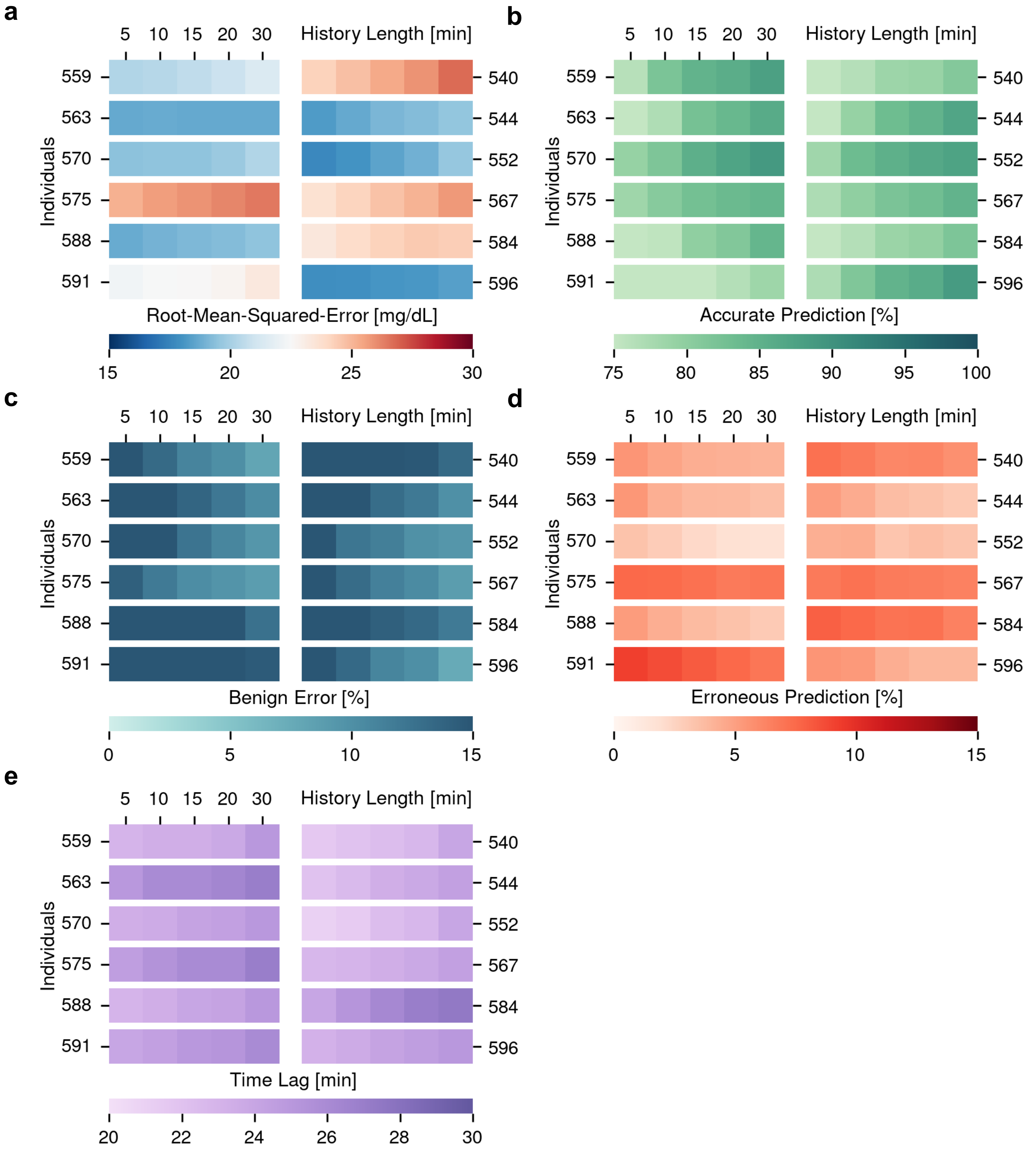


**Figure S3. Additional evaluation metrics on the analyses of different two-point history lengths.** Mean (5-fold cross-validation) (a) RMSE, percentage of (b) accurate predictions, (c) benign errors, (d) erroneous predictions and (e) time lag for a two point history length of 5, 10, 15, 20 and 30 minutes evaluated on the test sets of all individuals in the OhioTD1M dataset. With a less immediate gradients, blood glucose predictions become smoother favoring the CG-EGA evalutation metrics, in particularly the rate comparison between the true and predicted values. However, conversely the time lag increases as a less immediate gradient contains less information on the most recent blood glucose trends.

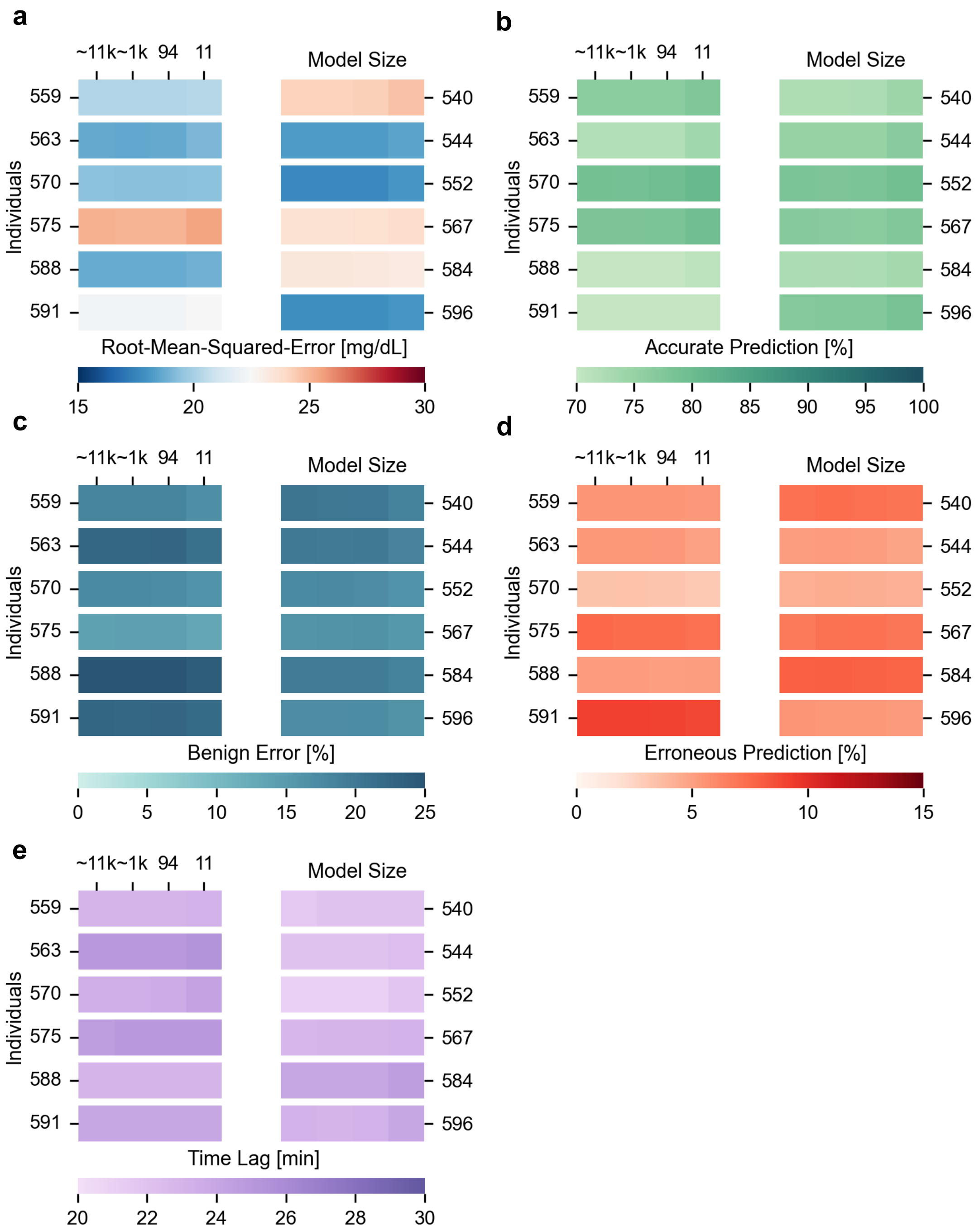


**Figure S4. Additional evaluation metrics on the analyses of reduced model sizes with a history length of 5 minutes.** Mean (5-fold cross-validation) (a) RMSE, percentage of (b) accurate predictions, (c) benign errors, (d) erroneous predictions and (e) time lag for model sizes [128, 64, 32, 16] (11265 parameters), [48, 16] (945 parameters), [9, 6] (94 parameters) and [2, 1] (11 parameters) evaluated on the test sets of all individuals in the OhioTD1M dataset. No performance loss is observed through all evaluated metrics other than the smallest model. For which, again, smoother transitions in the blood glucose predictions favor the CG-EGA evaluation metrics but not the mean time lag.

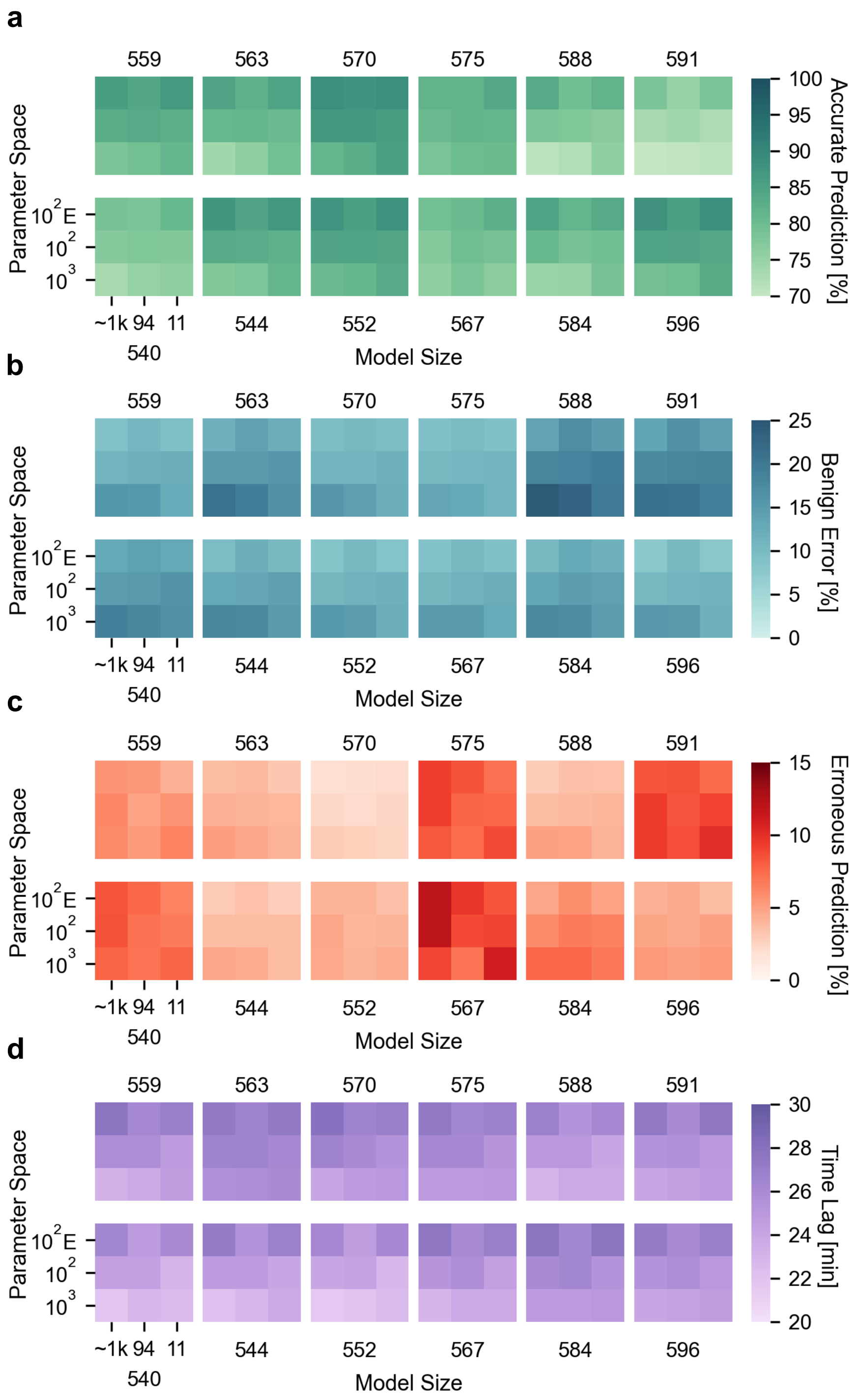


**Figure S5. Additional evaluation metrics on the analyses of the state reduction using limited parameter spaces.** Mean percentage of (a) accurate predictions, (b) benign errors, (c) erroneous predicions and (d) time lag over 5-cross validation folds for model sizes [48, 16] (945 parameters), [9, 6] (94 parameters) and [2, 1] (11 parameters) using a line-space of 1000, 100 and 100 ENODe (from measurements) states as possible parameter selections. The models are evaluated on the test sets of all individuals in the OhioTD1M dataset. Performance decreases with the introduction reduced state precision and increased non-linearity, however, highly dependent on the optimization of the hyperparameters.

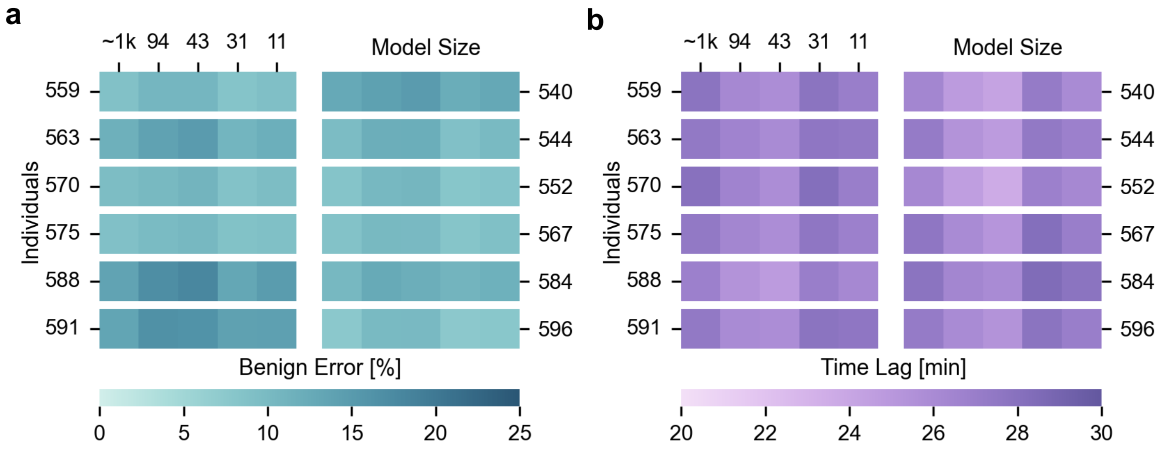


**Figure S6. Additional evaluation metrics on the analyses of the state reduction using ENODe limited parameter spaces.** Mean percentage of (a) benign errors and (b) time lag over 5-cross validation folds for model sizes [48, 16] (945 parameters), [9, 6] (94 parameters), [6, 3] (43 parameters), [4, 3] (31 parameters) and [2, 1] (11 parameters) using 100 ENODe (from measurements) states as possible parameter selection. The models are evaluated on the test sets of all individuals in the OhioTD1M dataset. Despite having the best RMSE over all individuals does model [9, 6] show worse performance according to the CG-EGA evaluation metrics. This is similar to model [6, 3] caused by more severe penalization of rate changes in the blood glucose predictions. The time lag does show to better for both the [9, 6] model as well as model [6, 3] as both are better optimized models.,

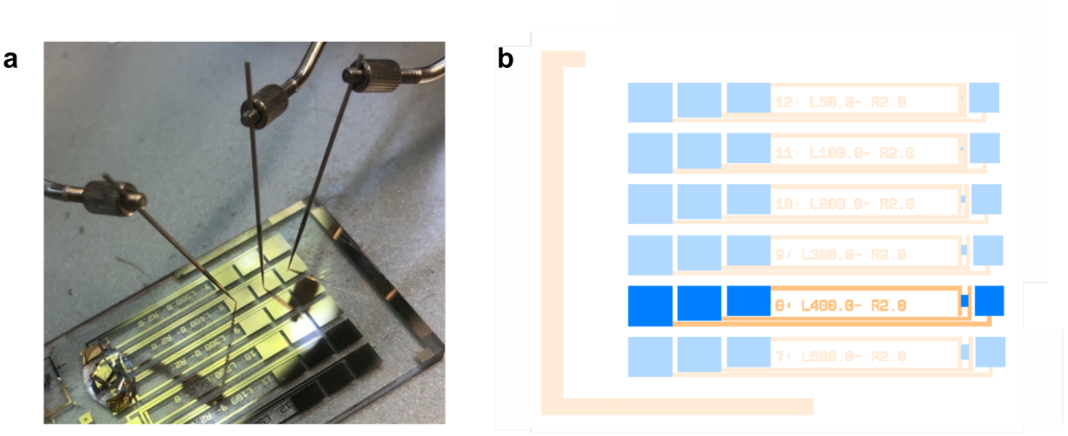


**Fig. S7. ENODe.** (a) Image of an ENODe and the measurement setup using needle probes. (b) Excerpt of the substrate layout containing multiple ENODes, relevant device is highlighted.

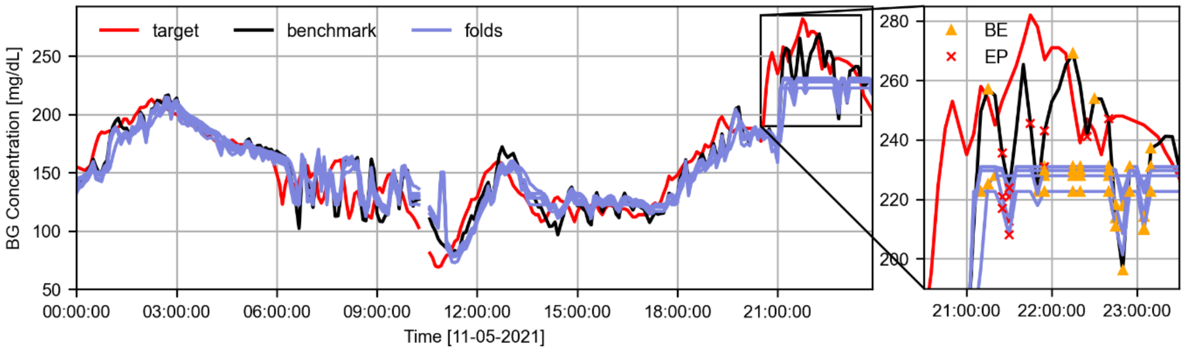


**Figure S8. Example of the real-time differences between the blood glucose predictions of the 5 models of the cross-validation folds of model [2, 1].** With target (red) the target data for individual 563 on the day 8 of the test set, benchmark (black) the predictions for the benchmark model with 180 minutes of history sampled at 5 minutes (software) and folds (purple) the cross-validation folds. The results show that all folds are capped in their glucose level reach for the smallest considered size.

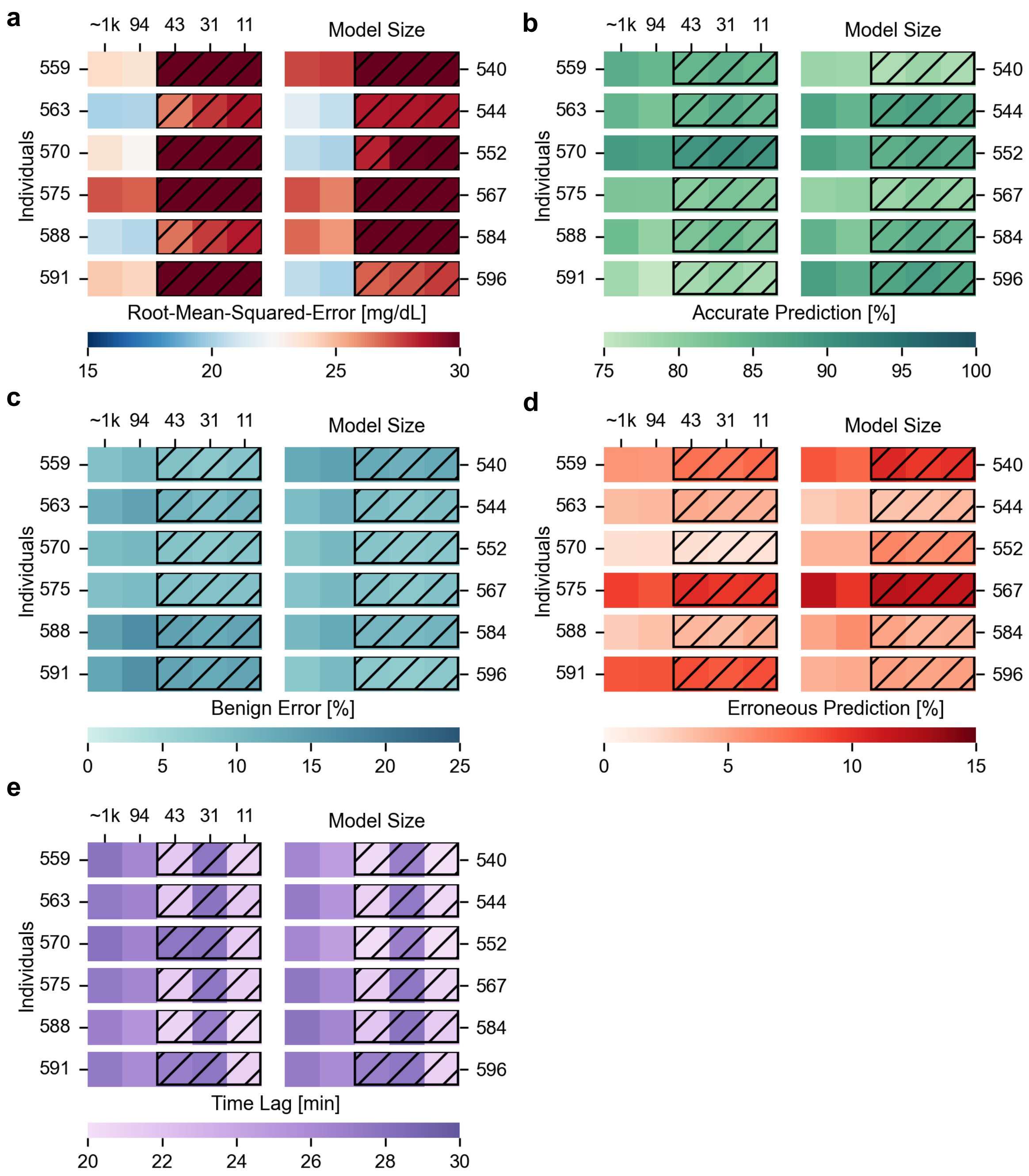


**Figure S9. Additional evaluation metrics on the analyses of the state reduction using ENODe limited parameter spaces without fixed model initializations.** Mean (a) RMSE, percentage of (b) accurate predictions, (c) benign errors, (d) erroneous predictions and (e) time lag over 5-cross validation folds for model sizes [48, 16] (945 parameters), [9, 6] (94 parameters), [6, 3] (43 parameters), [4, 3] (31 parameters) and [2, 1] (11 parameters) using 100 ENODe (from measurements) states as possible parameter selection. The models are evaluated on the test sets of all individuals in the OhioTD1M dataset. The models are trained with a fixed seed, nevertheless, the pseudo-random initializations cause some fold iterations to get stuck in local minima for sizes [6, 3], [4, 3] and [2, 1]. These stalled exceptions significantly influence the mean performance of the evaluated sizes (hashed) and are reconsidered with a different initialization technique.

| PF (min) | 5 min (SF) | 10 min (SF) | 15 min (SF) | 20 min (SF) | 30 min (SF) |
|---|---|---|---|---|---|
| 5 | 20.53 (2.25) | 21.59 (1.99) | 21.55 (2.23) | 21.56 (2.03) | 21.85 (2.1) |
| 10 | 20.46 (2.29) | 21.63 (2.01) | 21.44 (2.16) | 21.5 (2.05) | 21.85 (2.15) |
| 15 | 20.63 (2.21) | 21.48 (1.94) | 21.55 (2.25) | 21.54 (2.08) | 21.83 (2.13) |
| 20 | 20.65 (2.25) | 21.65 (1.96) | 21.51 (2.29) | 21.63 (2.19) | 21.85 (2.16) |
| 30 | 21.12 (2.4) | 21.73 (1.92) | 21.68 (2.24) | 21.65 (2.1) | 21.88 (2.18) |
| | **5 min (H)** | **10 min (H)** | **15 min (H)** | **20 min (H)** | **30 min (H)** |
| 5 | 20.74 (2.32) | 20.94 (2.43) | 21.06 (2.5) | 21.23 (2.56) | 21.59 (2.64) |
| | **~11k (S)** | **~1k (S)** | **94 (S)** | **11 (S)** | **-** |
| 5 | 20.74 (2.32) | 20.71 (2.31) | 20.73 (2.31) | 20.96 (2.38) | - |

**Table S1. Mean RMSE with standard deviation over the individuals of Cohort 2018 for the input and size reduction.** With PF the prediction frequency, SF the sampling frequency, H the history length and S the number of model parameters.

| PF (min) | 5 min (SF) | 10 min (SF) | 15 min (SF) | 20 min (SF) | 30 min (SF) |
|---|---|---|---|---|---|
| 5 | 21.49 (3.66) | 22.25 (3.98) | 22.24 (3.31) | 22.43 (3.25) | 23.1 (3.34) |
| 10 | 21.25 (3.19) | 22.3 (4.14) | 22.24 (3.44) | 22.49 (3.27) | 23.16 (3.38) |
| 15 | 21.44 (3.28) | 22.35 (4.04) | 22.26 (3.36) | 22.49 (3.26) | 23.17 (3.38) |
| 20 | 21.32 (3.27) | 22.4 (4.01) | 22.41 (3.42) | 22.62 (3.32) | 23.19 (3.35) |
| 30 | 21.95 (3.51) | 22.6 (4.12) | 22.55 (3.57) | 22.78 (3.46) | 23.24 (3.49) |
| | **5 min (H)** | **10 min (H)** | **15 min (H)** | **20 min (H)** | **30 min (H)** |
| 5 | 20.83 (2.93) | 21.23 (3.06) | 21.64 (3.14) | 21.96 (3.24) | 22.39 (3.33) |
| | **~11k (S)** | **~1k (S)** | **94 (S)** | **11 (S)** | **-** |
| 5 | 20.83 (2.93) | 21.23 (3.06) | 21.64 (3.14) | 21.96 (3.24) | - |

**Table S2. Mean RMSE with standard deviation over the individuals of Cohort 2020 for the input and size reduction.** With PF the prediction frequency, SF the sampling frequency, H the history length and S the number of model parameters.

| | | | | | |
|---|---|---|---|---|---|
| **LS1000** | **~1k (S)** | **96 (S)** | **-** | **-** | **11 (S)** |
| | 21.04 (2.38) | 21.07 (2.34) | - | - | 22.64 (2.29) |
| **LS100** | **~1k (S)** | **96 (S)** | **-** | **-** | **11 (S)** |
| | 22.41 (2.36) | 22.47 (2.37) | - | - | 23.18 (2.3) |
| **EN100** | **~1k (S)** | **96 (S)** | **-** | **-** | **11 (S)** |
| | 23.35 (2.54) | 22.97 (2.54) | - | - | 24.28 (2.6) |
| **EN100** | **~1k (S)** | **96 (S)** | **43 (S)** | **31 (S)** | **11 (S)** |
| | 23.35 (2.54) | 22.97 (2.54) | 23.09 (2.58) | 23.19 (2.59) | 24.28 (2.6) |

**Table S3. Mean RMSE with standard deviation over the individuals of Cohort 2018 for the state reduction.** With LS1000 the line-space of 1000 states, LS100 the line-space of 100 states, EN100 the 100 ENODe states and S the number of model parameters.

| | | | | | |
|---|---|---|---|---|---|
| **LS1000** | **~1k (S)** | **96 (S)** | **-** | **-** | **11 (S)** |
| | 21.05 (2.92) | 21.24 (2.87) | - | - | 23.01 (4.25) |
| **LS100** | **~1k (S)** | **96 (S)** | **-** | **-** | **11 (S)** |
| | 22.57 (3.06) | 22.86 (3.44) | - | - | 23.53 (4.36) |
| **EN100** | **~1k (S)** | **96 (S)** | **-** | **-** | **11 (S)** |
| | 24.07 (3.24) | 23.44 (3.57) | - | - | 25.13 (4.71) |
| **EN100** | **~1k (S)** | **96 (S)** | **43 (S)** | **31 (S)** | **11 (S)** |
| | 24.07 (3.24) | 23.44 (3.57) | 23.58 (3.42) | 24.02 (3.4) | 25.13 (4.71) |

**Table S4. Mean RMSE with standard deviation over the individuals of Cohort 2020 for the state reduction.** With LS1000 the line-space of 1000 states, LS100 the line-space of 100 states, EN100 the 100 ENODe states and S the number of model parameters.
